# Gender Medicine in Computed Tomography Radiomics Analysis to Predict Disease Progression in Liver Respectable Colorectal Cancer Patients

**DOI:** 10.1002/cam4.70991

**Published:** 2025-09-04

**Authors:** Annarita Fanizzi, Arianna Campione, Samantha Bove, Oronzo Brunetti, Deniz Can Guven, Angelo Cirillo, Andrea Lupo, Chiara Macrì, Leonardo Ricchitelli, Alessandro Rizzo, Elsa Vitale, Maria Colomba Comes, Raffaella Massafra

**Affiliations:** ^1^ Laboratorio Biostatistica e Bioinformatica IRCCS Istituto Tumori “Giovanni Paolo II” Bari Italy; ^2^ S.S.D. C.O.r.O. Bed Management Presa in Carico, TDM, IRCCS Istituto Tumori “Giovanni Paolo II” Bari Italy; ^3^ Department of Medical Oncology Hacettepe University, Cancer Institute Ankara Turkey; ^4^ Direzione Generale IRCCS Istituto Tumori “Giovanni Paolo II” Bari Italy; ^5^ Direzione Scientifica IRCCS Istituto Tumori “Giovanni Paolo II” Bari Italy

**Keywords:** cancer, colorectal liver metastases, computed tomography, gender medicine, machine learning, radiomics

## Abstract

**Background:**

Gender medicine is an evolving discipline that examines how diseases manifest and progress differently in men and women. Tailoring medical therapies and diagnostic approaches can enhance patient outcomes. While radiomics is emerging as a promising tool in personalized medicine, few studies evaluate its role in gender medicine within radiology. In this context, our preliminary objective was to determine whether radiomic features could predict disease‐free survival within 3 years after the last follow‐up in patients with colorectal liver metastases, with an emphasis on gender differences.

**Methods:**

The study analyzed preoperative CT scans of 196 patients from The Cancer Imaging Archive who underwent resection of colorectal cancer liver metastasis. Using the Pyradiomics library, we extracted 1316 features for each patient. We developed an analysis framework applied initially to the entire patient sample, then separately to male and female subsamples. This framework included: Volume of Interest (VOI) segmentation, handcrafted feature extraction and selection, detection of confounding patients, and training of ensemble classification models comprising five classifiers. Performance was assessed through 100 rounds of 10‐fold cross‐validation.

**Results:**

The selected feature subsets for male and female subsamples showed no overlap. The ensemble model demonstrated a notable improvement in performance when trained on the female subsample (mean AUC of 80.5%) compared to the model trained on the entire dataset (mean AUC of 64.8%), while performance for the male subsample remained nearly unchanged.

**Conclusion:**

Although further validation with a larger dataset and external confirmation is needed, these preliminary results suggest a meaningful impact of gender medicine in radiology.

## 1 Introduction

Gender Medicine is an emerging field focused on understanding how diseases manifest and progress differently between men and women. It recognizes that gender affects various aspects of health and medical treatment, including prevention strategies, clinical symptoms, diagnosis, therapeutic response, prognosis, and social and psychological impact [1, 2, 3, 4, 5]. The primary goal of gender medicine is to integrate gender differences into healthcare practices by considering the unique biological and physiological distinctions between men and women. Tailoring medical therapies and diagnostic approaches, as well as implementing gender‐specific prevention programs, can enhance patient outcomes.

This approach is particularly relevant in oncology, where evidence suggests that treatment responses and prognoses can differ significantly between genders [3, 4, 5]. Recent studies have highlighted that gender influences not only the incidence and mortality rates of various cancers but also clinical presentation and drug response.

In this context, radiological image analysis represents a new frontier in personalized oncological care [6, 7, 8, 9, 10]. Radiomics, supported by artificial intelligence, enables non‐invasive quantitative analysis of tissue characteristics directly from medical images. By extracting quantitative features from these images, radiomics provides insights into disease attributes such as aggressiveness, prognosis, and therapeutic response, which can be adapted to address gender‐specific differences. While radiomics has been applied across various medical conditions, its most advanced applications are in oncology. Quantitative features derived from medical images, including aspects such as shape, intensity, volume, size, and texture, provide detailed information about the tumor microenvironment and phenotype. Radiomics plays a complementary role in solving outstanding diagnostic and therapeutic challenges compared with information available from laboratory tests, clinical reports, or genomic/proteomic analyses. When combined with clinical data, these imaging features can be correlated with clinical outcomes, thereby supporting decision‐making in patient care. Radiomics thus offers a range of imaging biomarkers that can aid in cancer diagnosis, prognosis evaluation, treatment response prediction, and monitoring of disease progression [11].

However, much of the current literature overlooks gender as a variable when interpreting radiological data, limiting the potential of these tools to optimize patient care fully. Few studies have explored the role of gender medicine within clinical radiology [12].

In this study, our primary objective was to explore how gender medicine could be integrated into the analysis of radiological images in oncology. Specifically, we analyzed a case study to evaluate the potential of radiomics to predict disease progression within 3 years of follow‐up in patients with colorectal liver metastases (CRLM), assessing whether gender differences in radiomic features exist within this patient category. CRLM is considered a major problem after curative treatment and represents an important cause of colorectal cancer‐related death, since the liver is the most frequent organ of distant metastasis in this tumor [13]. Although practice‐changing progress in the development of novel anticancer agents has been recently made, understanding the mechanisms implied in CRLM is an unmet need in this setting. While prior studies have utilized radiomics in colorectal liver metastases for purposes such as predicting recurrence [14], disease‐specific survival [14], and overall survival [15], none have investigated whether a patient's gender might influence the predictive value of radiomic features and, consequently, the performance of machine learning models in classification or regression tasks.

To this end, we assessed the performance of machine learning models trained to predict disease progression based on radiomic features, first considering all patients together, then separately by gender. Machine learning models were subsequently trained and tested on three datasets: the full sample, male‐only, and female‐only.

## 2 Materials and Methods

### 2.1 Dataset Description

This study used a set of public radiological images extracted from the Cancer Imaging Archive (TCIA) referred to patients with colorectal liver metastases [16].

The dataset includes DICOM images and DICOM Segmentation Objects (DSOs) from 197 patients who underwent resection for colorectal cancer liver metastasis (CRLM). As described by the authors [16], masks corresponding to the liver, tumor, liver vessel, and portal segmentations, and the future liver remnant, were first saved in the ITK MetaImage format and then converted to DICOM segmentation objects (DSOs), in accordance with the Segmentation Information Object Definition. Quantitative image analysis was conducted on preoperative CT scans from this single‐institution cohort. Inclusion criteria required patients to have pathologically confirmed CRLM, preoperative portal venous contrast‐enhanced CT scans within 6 weeks of surgery, and available pathological data for both liver and tumor. Exclusion criteria included 90‐day mortality, less than 24 months of follow‐up, prior hepatic artery infusion chemotherapy, local tumor ablation, more than three wedge resections, or lack of visible tumor on imaging.

For patients who underwent neoadjuvant chemotherapy, post‐treatment/preoperative CT scans were analyzed. In cases of preoperative portal vein embolization (PVE), pre‐PVE CT scans were used, as PVE can alter liver parenchyma appearance in CT images and may impact hepatic enhancement patterns, which remain understudied.

The dataset also includes demographic, pathological, and survival data, supplemented by a data dictionary. For this study, disease‐free survival (DFS) in months was chosen as the outcome measure. For classification purposes, DFS was binarized based on a three‐year threshold: patients with DFS greater than 3 years were classified as positive cases, while those with DFS equal to or less than 3 years were classified as negative cases.

### 2.2 Image Pre‐Processing

**FIGURE 1:**
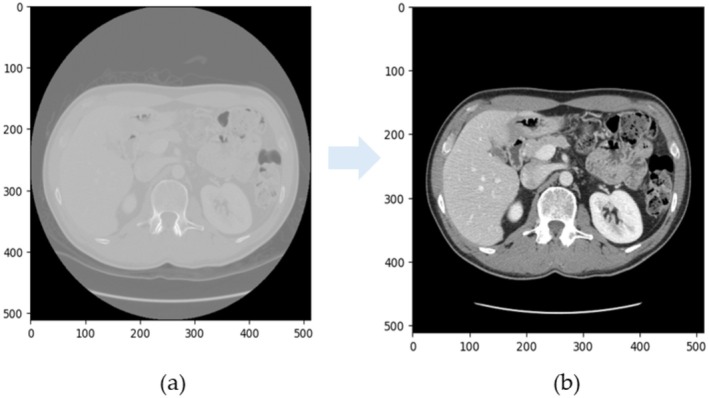
Example of a CT slice before (a) and after (b) contrast enhancement. Firstly, we convert raw pixel data values to Hounsfield Units; then we have adjusted the pixel values for visualization purposes.

In this study, the entire liver was designated as the region of interest (ROI), and images were filtered using liver masks extracted from the DSOs. CT scans were pre‐processed to enhance contrast in individual slices (Figure 1). Initially, raw pixel data values were converted to Hounsfield Units (HU) for meaningful clinical interpretation. The scale boundaries of the Hounsfield Units (HUs) were a range from 21.4 to 78.2. Next, pixel values were adjusted using a Value of Interest (VOI) Look‐Up Table, which enhances the display of specific pixel ranges (e.g., soft tissue or bone) by adjusting contrast and brightness.

The full sequence of slices was saved in Nearly Raw Raster Data format, with alignment of the origin, direction, and spacing of the associated masks to match the imaging parameters.

### 2.3 Feature Extraction With PyRadiomics

Feature extraction was conducted using PyRadiomics, a Python library widely used for radiomic feature analysis from medical images [17]. In this study, we enabled the extraction of multiple radiomic feature classes, including First‐Order Statistics (18 features), Shape‐based 3D (14 features), Gray Level Co‐occurrence Matrix (GLCM) (24 features), Gray Level Run Length Matrix (GLRLM) (16 features), Gray Level Size Zone Matrix (GLSZM) (16 features), Neighboring Gray Tone Difference Matrix (NGTDM) (5 features), and Gray Level Dependence Matrix (GLDM) (14 features).

First‐Order Statistics describe voxel intensity distributions within the ROI, independent of spatial relationships (e.g., mean, median, standard deviation). Shape‐Based Features capture geometric characteristics, such as volume and surface area. Texture Features, including GLCM, GLRLM, GLSZM, GLDM, and NGTDM, provide spatial relationships between voxel intensities, offering insight into texture. Except for shape features, each class of features was calculated on the original image and 13 derived images obtained by applying filters such as Wavelet (7 decompositions), Laplacian of Gaussian (LoG), Square, Square Root, Exponential, and Logarithm, resulting in a total of 1316 features per patient.

Feature extraction was customized by setting ‘binWidth’ to 17, ‘normalize’ to True, and ‘correctMask’ to True. No resampling was enabled as the origin, direction, and spacing parameters were previously set during contrast enhancement. The sigma parameter for the LoG filter was set to 1, emphasizing fine textures, while default settings were used for Wavelet decomposition, resulting in eight total decompositions.

### 2.4 Machine Learning Workflow: Feature Engineering and Prediction Models

**FIGURE 2:**
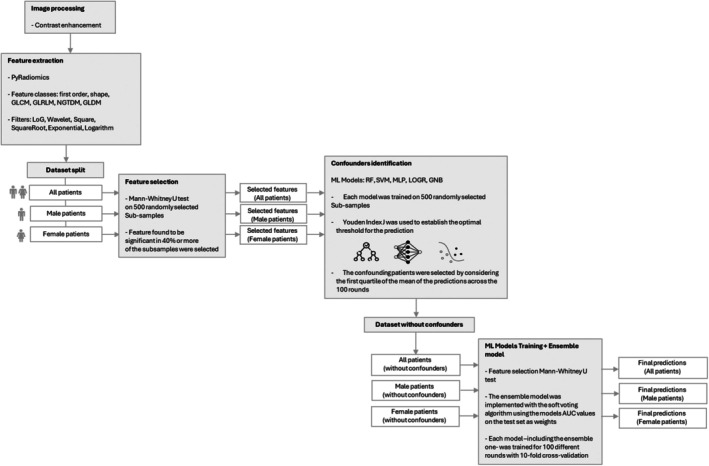
Analysis workflow. We defined a framework for analyzing patient images that was applied in a similar manner to both the entire patient sample (All dataset) and the two subsamples of male (Male dataset) and female (Female dataset) patients. The proposed analysis framework is developed in the following steps: (i) identification of a significant feature set; (ii) identification of confounding patients; (iii) training of classification models and performance evaluation.

The aim of this study was to assess the predictive power of radiomic features with gender‐specific differentiation. We developed an analytical framework applied first to the entire dataset (All dataset) and then to gender‐specific subsamples (Male dataset and Female dataset) (Figure 2). The framework was structured as follows: (i) identification of a significant feature set; (ii) detection of confounding cases; and (iii) classification model training and performance evaluation.

#### 2.4.1 Feature Selection Procedure

A feature selection process was performed on each patient set (all, male‐only, female‐only) [18]. Using filtering methods, significant features were selected through iterative Wilcoxon/Mann–Whitney testing on 500 randomly sampled subsets, each comprising 80% of the initial population. For each feature, the percentage of times it was found to be significant (*p* < 0.05) was calculated, and features significant in 40% or more of the samples were retained.

#### 2.4.2 Identification of Confounding Cases

The identification of confounding cases in a classification problem is a crucial step for improving the precision and reliability of a model. Confounding cases occur when a classifier struggles to correctly distinguish between two or more classes due to data overlapping, ambiguous features, or noise. These cases can lead to systematic errors, reducing the overall performance of the model and creating bias in the predictions. Recognizing and analyzing such cases not only allows for model refinement but also improves the understanding of the problem, thereby optimizing predictive accuracy and the system's effectiveness in handling complex situations. This step is even more important for the purposes of our work: identifying a sample free from bias or noise allows us to more clearly detect any gender differences in the radiomic features extracted from CT images.

To ensure the integrity of our results, we employed a systematic approach to identify these confounding samples: in each of the three datasets, five classifiers were trained on 500 random subsamples of 80% of the size of the sample to which they belong. For each of the three datasets, the distribution of the mean number of correct classifications in the 500 rounds and of the five classifiers was calculated; in each sample, patients whose mean misclassification exceeded the first quartile of said distribution were defined as confounders.

Following this identification process, we removed these patients from the dataset to mitigate their influence on the analysis.

The five machine learning algorithms used to predict the DFS were Random Forest (RF), Support Vector Machine (SVM), Multi‐Layer Perceptron (MLP), Logistic Regression (LogR), and Gaussian Naive Bayes (GNB). RF is an ensemble learning method that constructs multiple decision trees during training and outputs the class that is the mode of the classes (classification) or the mean prediction (regression) of the individual trees. RF is particularly robust to overfitting, especially in high‐dimensional datasets, due to its ability to average multiple decision trees, reducing variance [19].

SVM is a supervised learning model that finds the hyperplane that best separates data into different classes with the maximum margin [20]. In cases where data are not linearly separable, SVM uses a kernel trick to project the data into higher‐dimensional space to make it linearly separable. SVM is known for its effectiveness in high‐dimensional spaces and its ability to handle non‐linear boundaries, though it can be computationally expensive for large datasets.

MLP is a type of artificial neural network consisting of an input layer, one or more hidden layers, and an output layer [21]. Each neuron in a layer is connected to every neuron in the following layer. MLP learns by adjusting weights through backpropagation, which minimizes the prediction error. MLP is a versatile model capable of learning complex non‐linear relationships, though it requires careful tuning of hyperparameters such as the number of hidden layers and neurons.

LogR is a simple and interpretable model often used for binary classification [22]. It estimates the probability that a given input belongs to a particular class by fitting data to a logistic function. While it assumes a linear relationship between the input features and the log‐odds of the output, it performs well when the true decision boundary is indeed linear or nearly so.

GNB is a probabilistic classifier based on Bayes' Theorem with the assumption that the features are conditionally independent given the class [23]. GNB assumes that each feature follows a Gaussian distribution. Despite its simplicity, GNB is computationally efficient and works well with high‐dimensional data, although its performance might degrade if the independence assumption is strongly violated.

#### 2.4.3 Ensemble Machine Learning Model

After removing confounding patients, we trained five classification models, such as RF, SVM, MLP, LogR, and Gaussian (GNB). Then, an ensemble model was finally constructed using the *Soft Voting Classifier* method [24, 25]. The underlying concept of the *Soft Voting Classifier* is to combine conceptually distinct machine learning classifiers and employ either majority voting (max voting) or the average of predicted probabilities (soft voting) to determine the outcome.

Specific weights can be assigned to each classifier. When weights are provided, the predicted probabilities for each classifier are computed, multiplied by the respective classifier weight, and averaged. The final label is derived from the label with the highest average probability.

In this case, soft voting was performed and the AUC values—each for the individual model considered—were used as weights. Thus, a higher AUC results in a greater weight for the corresponding model in the voting process.

For each dataset, the classification performances of all machine learning models and ensemble models were evaluated on 100 10‐fold cross‐validation rounds.

To evaluate the performance of the classifiers, the following metrics were considered: accuracy, precision, sensitivity, specificity, F1 score, and area under the curve (AUC). For each model, the Youden Index J was utilized to establish the optimal threshold for the prediction [26].

#### 2.4.4 Statistic Analysis

All the classification performances of the radiomic‐based models were evaluated in terms of mean and confidence interval 95%.

To evaluate the statistical association between clinical features and the binary interest outcome, we used Chi‐Square when the clinical feature was measurable on a nominal scale and Wilcoxon Mann–Whitney test when the clinical feature was measurable on an interval scale. Moreover, to verify that the distributions of individual clinical variables in the subsample of men are significantly equal to those of women, we used the non‐parametric Wilcoxon Mann–Whitney test when the clinical feature was measurable on an interval scale and Chi‐Square when the clinical feature was measurable on a nominal scale.

Using a *T*‐Student test, the classification metrics related to the model trained on all dataset were compared with that obtained from training the model on male and female. Finally, we used the method by Hanley and McNeil [27, 28] to compare the AUC mean value of the different models.

A result was considered statistically significant when the *p*‐value was less than 0.05.

## 3 Results

### 3.1 Characteristics of Dataset

Table 1 summarizes the characteristics of the patients analyzed. A total of 196 patients with a median age of 61 (1st–3rd quartiles of 52–69) were included in the study. Among these, 117 patients (59.7%) experienced disease progression within 3 years (positive cases).

**TABLE 1:** Patients' characteristics. The distributions of clinical characteristics in both the overall sample and the two sub‐samples (male and female) are shown.

Characteristics	All patients (196 patients)	Male patients (117 patients)	Female patients (79 patients)
Disease progression within 3 years			
Positive (Abs; %)	117; 59.7%	70 [59.8%]	48 [60.8%]
Negative (Abs; %)	79; 40.3%	47 [40.2%]	31 [39.3%]
Age			
Median (1st quartile; 3rd quartile)	61 (52; 69)	61 (52; 69)	61 (52; 68.75)
Node primary			
Positive (Abs; %)	68; 34.69%	48; 41.03%	20; 25.32%
Negative (Abs; %)	128; 65.30%	69; 58.97%	59; 74.68%
Nan (Abs; %)	—		—
Synchronous CRLM			
Positive (Abs; %)	111; 56.63%	65; 55.56%	46; 58.23%
Negative (Abs; %)	85; 43.37%	52; 44.46%	33; 41.77%
Nan (Abs; %)	—	—	—
Carcinoembryonic antigen			
Median (1st quartile; 3rd quartile)	5.35 (2.48; 11.98)	5.35 (2.38; 11.93)	5.2 (2.15; 11.40)
Nan (Abs; %)	10	6	4
Total response percent			
Median (1st quartile; 3rd quartile)	0.50 (0.35; 0.70)	0.50 (0.35; 0.70)	0.50 (0.35; 0.70)
Nan (Abs; %)	3; 1.53%	3; 2.56%	—

In the full sample, 117 patients (59.7%) were men, and 79 (40.3%) were women. Disease progression within 3 years was observed in 70 men (59.8%) and 48 women (60.8%).

To verify that gender differences in radiomic feature predictive power were not influenced by other sample characteristics, statistical evaluations were performed. The distributions of men's and women's characteristics do not differ statistically from each other, except for node primary status(*p* < 0.05). Moreover, no clinical characteristics were significantly associated with the primary outcome.

### 3.2 Results of Features Selection Procedure

**FIGURE 3:**
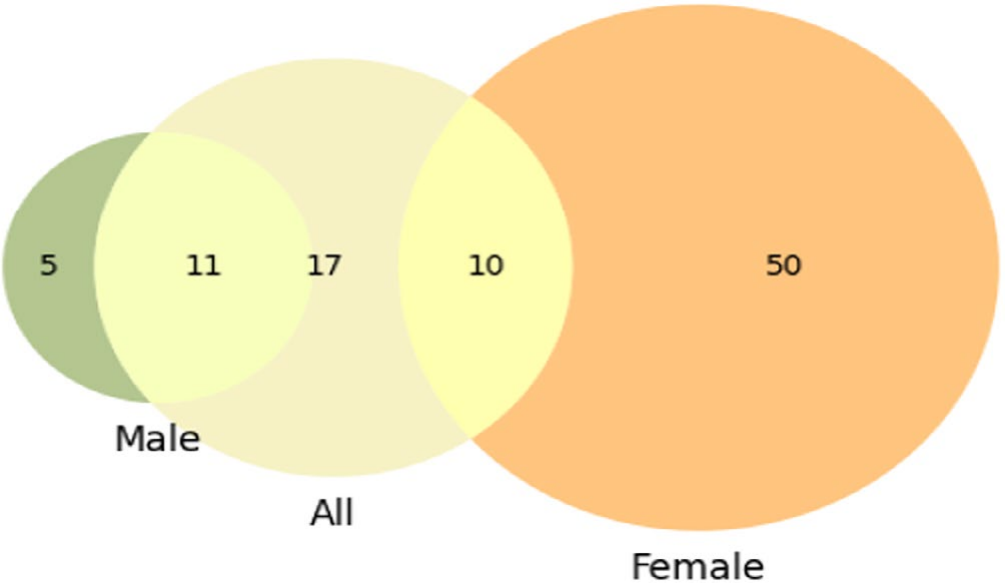
Feature selected for each sample dataset. A filtering procedure was implemented to identify significant features for each sample dataset. A total of 38 significant features were selected from the entire dataset. No feature was significant for both the subsample of men and women.

From the full dataset, 38 significant features were identified. Within the male and female subsamples, 16 and 60 significant features were identified, respectively (Figure 3). Notably, there was no overlap of significant features between the male and female subsamples. Of the 16 significant features identified in the male subsample, 11 were also significant in the full dataset, whereas only 10 of the 60 significant features from the female subsample overlapped with those in the full dataset (Table 2).

**TABLE 2:** Selected features in each sample set (all, male, female). In the first Column, in black are indicated the features selected for the all dataset only, in green and red are indicated the features that were also selected for the male and female sample, respectively. In the second and third Column, green and red indicate the features selected only for male and female, respectively.

**All** original_gldm_LargeDependenceLowGrayLevelEmphasis wavelet‐LHL_glcm_ClusterProminence wavelet‐LHL_glcm_ClusterTendency wavelet‐LHL_glcm_Contrast wavelet‐LHL_glcm_Correlation wavelet‐LHL_glcm_DifferenceAverage wavelet‐LHL_glcm_Id wavelet‐LHL_glcm_Idm wavelet‐LHL_glcm_Idmn wavelet‐LHL_glcm_Idn wavelet‐LHL_glcm_Imc2 wavelet‐LHL_glcm_InverseVariance wavelet‐LHL_glcm_MCC wavelet‐LHL_glcm_MaximumProbability wavelet‐LHL_glcm_SumEntropy wavelet‐LHL_glszm_GrayLevelNonUniformity wavelet‐LHL_glszm_SizeZoneNonUniformity wavelet‐LHL_ngtdm_Complexity wavelet‐LHL_ngtdm_Contrast wavelet‐LHH_glrlm_GrayLevelNonUniformityNormalized wavelet‐LHH_glrlm_GrayLevelVariance wavelet‐HLL_glcm_Autocorrelation wavelet‐HHL_firstorder_Median wavelet‐HHL_glcm_ClusterShade wavelet‐HHL_glcm_JointAverage wavelet‐HHL_glcm_SumAverage wavelet‐HHL_gldm_HighGrayLevelEmphasis wavelet‐HHL_gldm_LowGrayLevelEmphasis wavelet‐HHL_glszm_HighGrayLevelZoneEmphasis wavelet‐HHL_glszm_LowGrayLevelZoneEmphasis wavelet‐LLL_gldm_LargeDependenceLowGrayLevelEmphasis log‐sigma‐1‐mm‐3D_firstorder_Kurtosis log‐sigma‐1‐mm‐3D_gldm_LargeDependenceHighGrayLevelEmphasis log‐sigma‐1‐mm‐3D_gldm_SmallDependenceEmphasis log‐sigma‐1‐mm‐3D_gldm_SmallDependenceHighGrayLevelEmphasis log‐sigma‐1‐mm‐3D_glrlm_ShortRunHighGrayLevelEmphasis squareroot_gldm_LargeDependenceLowGrayLevelEmphasis logarithm_gldm_LargeDependenceLowGrayLevelEmphasis	**Male** wavelet‐LHH_glszm_SmallAreaHighGrayLevelEmphasis wavelet‐HLH_glrlm_GrayLevelNonUniformityNormalized wavelet‐HLH_glrlm_GrayLevelVariance wavelet‐HHH_glszm_HighGrayLevelZoneEmphasis wavelet‐HHH_glszm_LowGrayLevelZoneEmphasis **Female** original_shape_Maximum2DDiameterColumn original_shape_Maximum2DDiameterRow original_shape_MinorAxisLength wavelet‐LHL_glcm_MaximumProbability wavelet‐LHL_glcm_SumEntropy wavelet‐LHH_firstorder_10Percentile wavelet‐LHH_firstorder_90Percentile wavelet‐LHH_glcm_SumSquares wavelet‐HLL_firstorder_Kurtosis wavelet‐HLL_glcm_ClusterProminence wavelet‐HLL_glcm_ClusterTendency wavelet‐HLL_glcm_Contrast wavelet‐HLL_glcm_Correlation wavelet‐HLL_glcm_DifferenceAverage wavelet‐HLL_glcm_Id wavelet‐HLL_glcm_Idm wavelet‐HLL_glcm_Idmn wavelet‐HLL_glcm_Idn wavelet‐HLL_glcm_Imc2 wavelet‐HLL_glcm_InverseVariance wavelet‐HLL_glcm_MCC wavelet‐HLL_gldm_DependenceEntropy wavelet‐HLL_gldm_DependenceVariance wavelet‐HLL_ngtdm_Complexity wavelet‐HLL_ngtdm_Contrast wavelet‐HLH_firstorder_Kurtosis wavelet‐HLH_glcm_ClusterShade wavelet‐HLH_glcm_JointAverage wavelet‐HLH_glcm_SumAverage	wavelet‐HLH_glcm_SumSquares wavelet‐HHL_firstorder_10Percentile wavelet‐HHL_firstorder_90Percentile wavelet‐HHL_firstorder_InterquartileRange wavelet‐HHL_firstorder_MeanAbsoluteDeviation wavelet‐HHL_firstorder_RobustMeanAbsoluteDeviation wavelet‐HHH_firstorder_10Percentile wavewavelet‐HHH_firstorder_InterquartileRange wavelet‐HHH_firstorder_MeanAbsoluteDeviation wavelet‐HHH_firstorder_RobustMeanAbsoluteDeviation wavelet‐LLL_glcm_Correlation wavelet‐LLL_glcm_MCC wavelet‐LLL_glrlm_HighGrayLevelRunEmphasis wavelet‐LLL_glrlm_LowGrayLevelRunEmphasis wavelet‐LLL_ngtdm_Strength log‐sigma‐1‐mm‐3D_firstorder_Energy log‐sigma‐1‐mm‐3D_firstorder_Median log‐sigma‐1‐mm‐3D_firstorder_TotalEnergy log‐sigma‐1‐mm‐3D_glcm_ClusterTendency log‐sigma‐1‐mm‐3D_glszm_GrayLevelNonUniformity let‐HHH_firstorder_90Percentile

Most of the significant features were second‐order features derived from images processed with wavelet transforms or LoG filters.

### 3.3 Identification of Confounding Patients

**FIGURE 4:**
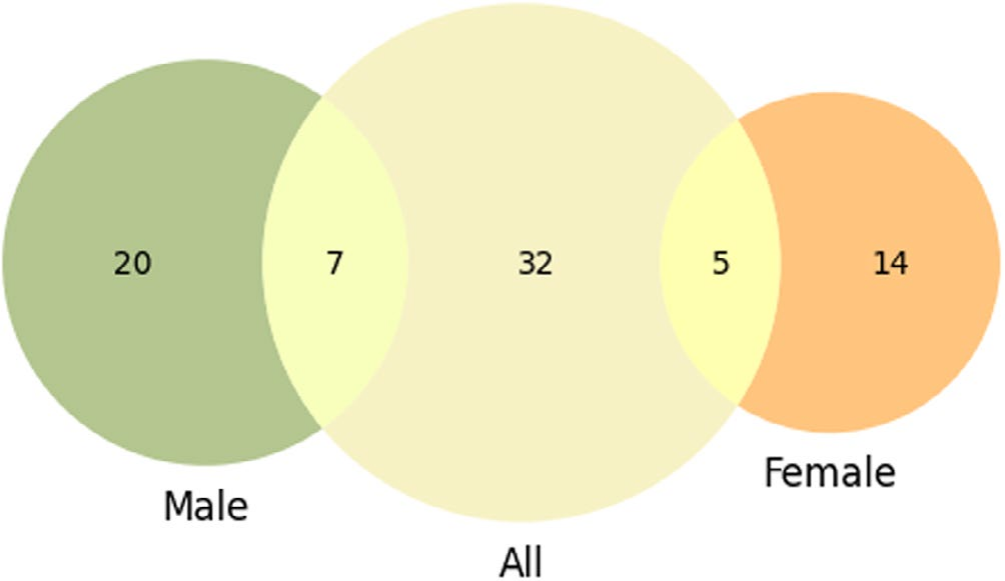
Confounder patients distribution. Number of confounder patients identified by the recursive procedure for all sample patients, and male and female sub‐sample.

Five classifiers were trained on 500 randomly selected subsets comprising 80% of the reference dataset size. For each dataset (all, male, female), the distribution of correct classifications across the 500 rounds was calculated. Patients whose mean misclassification rate exceeded the first quartile of this distribution were classified as confounding cases. In the full dataset, 44 confounding patients (22.44%) were identified, while 27 (23.08%) and 19 (24.05%) confounding cases were identified in the male and female subsamples, respectively (Figure 4). Table 3 shows the distribution of positive and negative cases before and after confounding patients were removed from each dataset.

**TABLE 3:** Distribution of patients with respect to the outcome of interest, with and without confounders. In each sample and sub‐sample, after removal of confounding patients, the proportions of patients who experience disease progression (positive cases) and those who do not experience disease progression (negative cases) are comparable with the starting distributions.

	All patients		Male patients		Female patients	
	With confounders	Without confounders	With confounders	Without confounders	With confounders	Without confounders
Total number	196	158	117	89	79	64
Positive	117 [59.7%]	95 [60.1%]	70 [59.8%]	57 [64%]	48 [60.8%]	42 [65.6%]
Negative	79 [40.3%]	63 [39.9%]	47 [40.2%]	32 [36%]	31 [39.3%]	22 [34.4%]

### 3.4 Classification Performances

**FIGURE 5:**
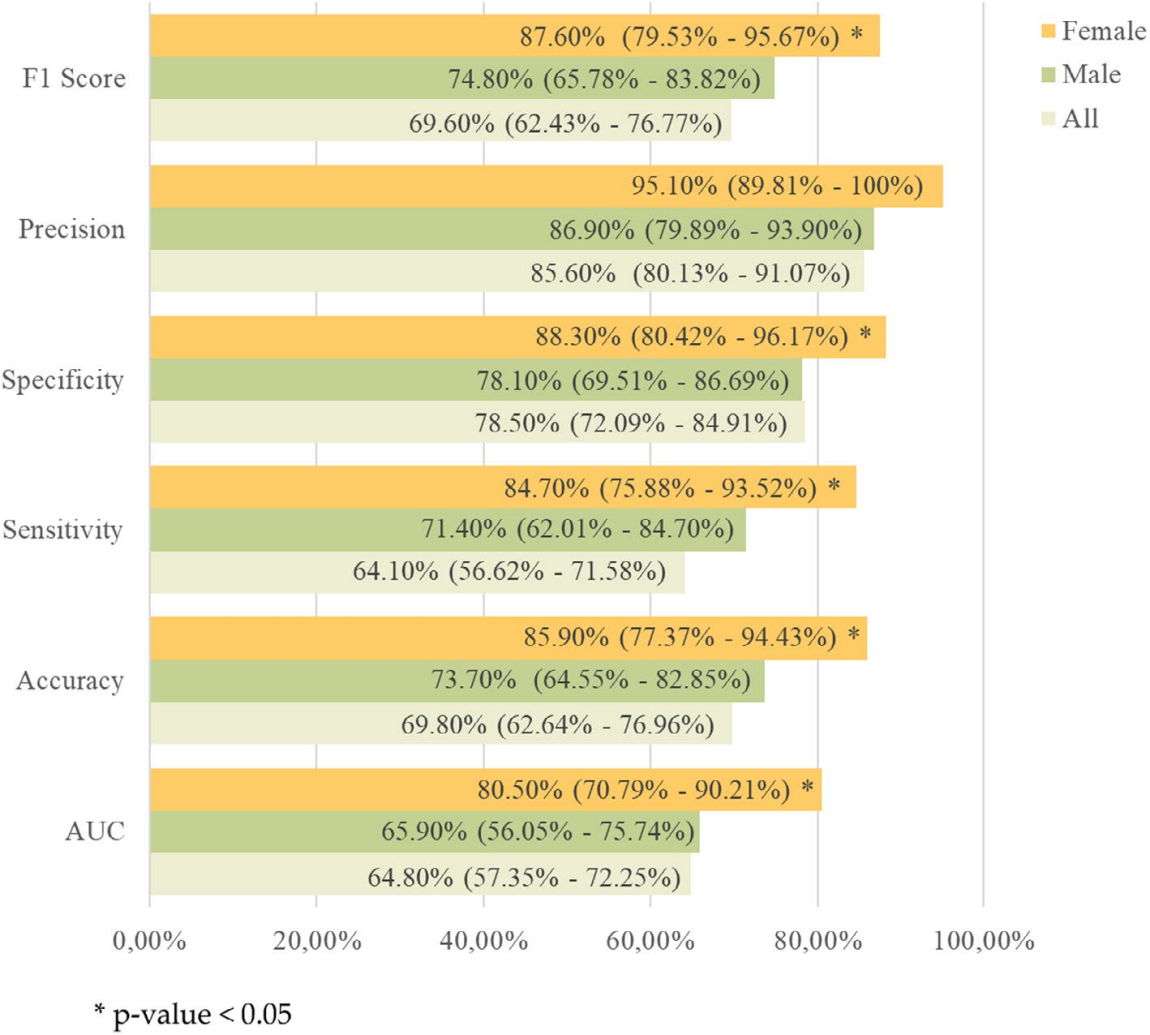
Classification performances achieved on 100 10‐fold cross‐validation schemas by the ensemble radiomic‐based models trained on all, male, and female datasets. The mean value and 95% Confidence Interval (95% CI) are reported in percentage values. *p < 0.05.

The classification performance metrics, averaged over 100 rounds of 10‐fold cross‐validation, are presented in Figure 5, along with 95% confidence intervals.

The model trained separately on male and female datasets achieved an average AUC of 65.9% and 80.5%, respectively, with corresponding accuracies of 73.7% and 85.9%, and F1 scores of 74.8% and 87.6%. In comparison, the model trained on the entire dataset achieved an average AUC of 64.8%, accuracy of 69.8%, and F1 score of 69.6%.

The ensemble model demonstrated a significant increase in performance when trained on the female dataset, as compared to the full dataset; in contrast, the model trained on the male dataset showed performance metrics similar to those achieved on the entire dataset.

## 4 Discussion

Gender medicine is rapidly emerging as a field dedicated to understanding how gender influences the prevention, prognosis, and diagnosis of various diseases. Recognizing and incorporating these differences into healthcare can support individualized care by facilitating personalized therapies, gender‐specific diagnostic and therapeutic pathways, and targeted prevention initiatives. The goal is to learn from gender differences to improve care for both men and women [5].

In recent years, researchers have highlighted the importance of including sex and gender analyses in all phases of research [29]. Literature increasingly points to gender differences as key factors in symptom presentation, access to treatment, and the occurrence of severe side effects, particularly in fields like cardiology and pharmacology [30, 31, 32]. However, the role of gender in oncology—with respect to incidence, prognosis, mortality, and treatment response—remains largely underexplored [5].

The concept of gender medicine aligns with the current emphasis on personalized medicine, with radiological imaging representing a new frontier in this area. Radiomics, defined as the automated extraction of quantitative morphological and textural features from images, has shown significant value, particularly in oncology. Radiomics provides information on disease type, prognosis, and treatment response directly from images [6, 7, 8, 9, 10]. Artificial intelligence systems are being developed to address crucial clinical needs based on radiological image analysis. Importantly, these systems must be fair and unbiased, as research has shown that artificial intelligence can inherit and even amplify human biases, such as gender or racial biases [33, 34, 35]. This tendency of artificial intelligence systems to learn distorted patterns is particularly dangerous in the context of healthcare [29, 36]. Although efforts to develop accurate diagnostic and prognostic algorithms are substantial, the impact of gender on automated predictive models remains largely overlooked in medical imaging research [37, 38, 39].

In this scenario, this study aimed to explore the impact of gender differences in clinical radiology, focusing on prognosis prediction in patients with resectable colorectal liver metastases (CRLM) based on radiomic features extracted from preoperative CT scans. While acknowledging that gender is a non‐binary construct, this study was limited to a male–female analysis.

Colorectal cancer, a common malignancy of the digestive system, frequently metastasizes to the liver via the portal vein [11, 14, 40]. Several radiomic studies have aimed to diagnose and predict prognosis in CRLM patients using CT imaging [41, 42, 43]. After interventions like surgical resection, radiofrequency ablation, and targeted chemotherapy, some CRLM patients achieve long‐term survival, while others may only reach a limited tumor‐free survival. Given these variations, personalized treatment strategies are crucial for developing appropriate and individualized surveillance plans.

The preliminary results of our study suggest a significant gender‐based differentiation in the radiomic features predictive of disease progression within 3 years. Specifically, there was minimal overlap in statistically significant features between male and female samples; the significant feature sets differed substantially between men and women. The male dataset identified only 16 significant features, while the female dataset identified 50. Additionally, the ensemble model showed improved performance in the female dataset, with an increase of approximately 15 percentage points in AUC compared to the model trained on the full dataset. In contrast, the model trained on the male dataset achieved results similar to those of the full dataset.

Liver radiomic differences between men and women result from various biological mechanisms. Estrogen reduces fibrosis and affects vascularization, whereas testosterone may increase steatosis. The female liver has a greater immune response, affecting inflammation and fibrosis. Genetic and epigenetic differences regulate metabolism and tissue repair. In addition, variations in perfusion and angiogenesis affect oxygenation and liver structure.

Literature suggested the possibility of radiomics to predict disease progression and its related treatment response [9, 44]. In this regard, Andersen et al. [45] suggested a correlation existed between homogeneity features and overall survival (OS). On the other hand, heterogeneity features could predict worse OS, respectively [46]. Moreover, the importance of radiomic features in the cancer‐related outcomes has been highlighted [42, 46, 47]. However, future studies will be needed to better assess radiomic features with cancer progression. In this scenario, this study represented a novelty, since we considered feature characterizations according to gender differences.

Our study highlights that radiomic features predictive of tumor progression differ between men and women, suggesting the importance of gender‐specific analyses in personalized medicine. This approach improves diagnostic accuracy, optimizes therapies, and reduces bias in artificial intelligence models. Gender medicine in radiomics represents a crucial step toward more equitable and personalized care. Gender‐specific predictive models could refine therapeutic selection and risk stratification. The use of gender‐based radiomic biomarkers would promote more accurate monitoring of tumor progression. It could also optimize follow‐up protocols and assessment of response to treatments.

Although statistical checks were performed to ensure that the observed results were not biased by differences in clinical characteristics between male and female samples, there may still be biases introduced by the retrospective nature of the sample selection. Consequently, it is essential to acknowledge the limitations of this study, including sample size and the use of a public dataset.

Therefore, the proposed analyses require further validation studies, including prospective data cohorts that allow for the selection of male–female sub‐samples free from clinical‐demographic biases, and some biases should be acknowledged, including the heterogeneity of patient population and the lack of molecular and histopathological data. Additionally, a model validation on external datasets will ensure the robustness of our models. To validate our results on external datasets, we plan to test the developed models on independent cohorts from other oncology institutions, ensuring diversity in data and imaging protocols. In addition, we will use image harmonization techniques to reduce acquisition‐related variations. These steps will strengthen the reliability and generalizability of the results and the clinical applicability of radiomics in precision gender medicine. Finally, it should be emphasized that, the use of different imaging modalities could affect the generalizability of our results because of differences in the radiomic features extracted from each technique. For example, magnetic resonance imaging (MRI) and positron emission tomography (PET) offer functional and metabolic information that could improve the predictivity of models compared with CT alone. However, variability in acquisition protocols and technical parameters could introduce heterogeneity into the data. To address this challenge, future studies will evaluate the reproducibility of radiomic features on multimodal and standardized datasets.

## 5 Conclusion

Our study aligns with and expands upon existing literature on gender differences in cancer prognosis and treatment response. Prior research has shown that sex influences cancer incidence, progression, and therapeutic outcomes, with women often exhibiting stronger immune responses and different drug metabolism profiles. Studies in oncology, particularly in immunotherapy and chemotherapy, highlight gender‐based variations in efficacy and toxicity. However, few works have explored gender differences in radiomics. Our preliminary results suggest that radiomic features predictive of disease progression differ significantly between men and women, with improved performance of the predictive model in the female subgroup. The absence of overlap in selected features between the two groups strengthens the hypothesis that biological gender differences may influence the quantitative representation of disease in radiological images.

These results underscore the importance of considering gender in the development of artificial intelligence models for precision medicine to improve diagnostic accuracy and personalization of therapeutic strategies. Integrating gender into radiological analysis could also have a positive impact on other diseases.

However, further validation on larger, independent cohorts is needed to confirm these findings and further investigate the role of radiomic characteristics in personalizing cancer treatment. Future research will need to integrate molecular and clinical data to refine the understanding of the interactions between gender, radiomic imaging, and therapeutic response, thus contributing to increasingly personalized and inclusive medicine.

## Author Contributions

**Arianna Campione:** conceptualization, methodology, software, data curation, validation, formal analysis, writing – original draft, writing – review and editing. **Samantha Bove:** writing – review and editing, software, formal analysis. **Oronzo Brunetti:** writing – review and editing. **Deniz Can Guven:** writing – review and editing. **Angelo Cirillo:** writing – review and editing. **Andrea Lupo:** writing – review and editing. **Chiara Macrì:** writing – review and editing. **Leonardo Ricchitelli:** writing – review and editing. **Alessandro Rizzo:** writing – original draft, writing – review and editing. **Elsa Vitale:** writing – original draft, writing – review and editing. **Maria Colomba Comes:** software, formal analysis, writing – original draft, writing – review and editing. **Raffaella Massafra:** conceptualization, resources, writing – review and editing, formal analysis. **Annarita Fanizzi:** conceptualization, methodology, software, validation, formal analysis, data curation, writing – original draft, writing – review and editing.

## Ethics Statement

The authors have nothing to report.

## Consent

The authors have nothing to report.

## Conflicts of Interest

The authors declare no conflicts of interest.

## Data Availability

The data was obtained from the open‐access NSCLC‐Radiogenomics dataset publicly available at the Cancer Imaging Archive (TCIA) database (https://www.cancerimagingarchive.net/collection/colorectal‐liver‐metastases/).
